# Associations between neurovascular coupling and cerebral small vessel disease: A systematic review and meta-analysis

**DOI:** 10.1177/23969873231196981

**Published:** 2023-09-11

**Authors:** Sheng Yang, Alastair John Stewart Webb

**Affiliations:** Wolfson Centre for Prevention of Stroke and Dementia, Nuffield Department of Clinical Neurosciences, University of Oxford, Oxford, UK

**Keywords:** Blood-oxygen-level-dependent (BOLD), cerebral small vessel disease, endothelial function, functional neuroimaging, neurovascular coupling

## Abstract

**Purpose:**

The pathogenesis of cerebral small vessel disease (cSVD) remains elusive despite evidence of an association between white matter hyperintensities (WMH) and endothelial cerebrovascular dysfunction. Neurovascular coupling (NVC) may be a practical alternative measure of endothelial function. We performed a systematic review of reported associations between NVC and cSVD.

**Methods:**

EMBASE and PubMed were searched for studies reporting an association between any STRIVE-defined marker of cSVD and a measure of NVC during functional magnetic resonance imaging, transcranial Doppler, positron emission tomography, near-infrared spectroscopy or single-photon emission computed tomography, from inception to November 3rd, 2022. Where quantitative data was available from studies using consistent tests and analyses, results were combined by inverse-variance weighted random effects meta-analysis.

**Findings:**

Of 29 studies (19 case-controls; 10 cohorts), 26 reported decreased NVC with increasing severity of cSVD, of which 18 were individually significant. In 28 studies reporting associations with increasing WMH, 25 reported reduced NVC. Other markers of cSVD were associated with reduced NVC in: eight of nine studies with cerebral microbleeds (six showing a significant effect); three of five studies with lacunar stroke; no studies reported an association with enlarged perivascular spaces. Specific SVD diseases were particularly associated with reduced NVC, including six out of seven studies in cerebral amyloid angiopathy and all four studies in CADASIL. In limited meta-analyses, %BOLD occipital change to a visual stimulus was consistently reduced with more severe WMH (seven studies, SMD −1.51, *p* < 0.01) and increasing microbleeds (seven studies, SMD −1.31, *p* < 0.01).

**Discussion and Conclusion:**

In multiple, small studies, neurovascular coupling was reduced in patients with increasing severity of all markers of cSVD in sporadic disease, CAA and CADASIL. Cerebrovascular endothelial dysfunction, manifest as impaired NVC, may be a common marker of physiological dysfunction due to small vessel injury that can be easily measured in large studies and clinical practice.

## Graphical abstract

**Graphical abstract:**
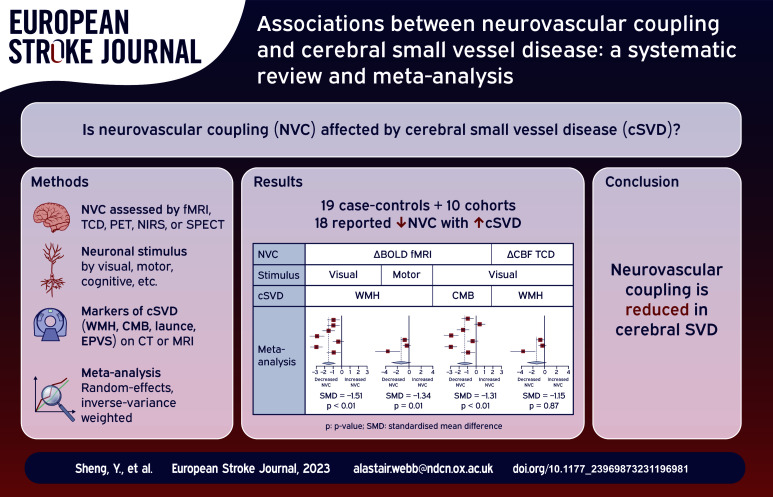

## Introduction

Cerebral small vessel disease (cSVD) accounts for 30% of ischaemic stroke, 80% of haemorrhagic stroke and 40% of dementia.^1^ Structural features of cSVD on magnetic resonance imaging (MRI) or computer tomography (CT) are well characterized, including white matter hyperintensities (WMH), lacunes of presumed vascular origin (lacunes), cerebral microbleeds (CMB), enlarged perivascular spaces (EPVS) and cerebral atrophy, but the underlying pathophysiology and mechanisms of cognitive dysfunction are unclear.^2^ However, both imaging markers of cSVD and clinical outcomes are strongly associated with impaired endothelial function,^3^ which is a key target of current trials in cSVD. The LACunar Intervention Trial-2 (LACI-2) demonstrated potential reduced cognitive decline with two endothelial-stabilizing drugs (cilostazol and isosorbide mononitrate) in cSVD patients, particularly with isosorbide mononitrate.^4,5^ Similarly, the Oxford haemodynamic adaptation to reduce pulsatility (OxHARP) trial is testing the potential of sildenafil, a PDE5 inhibitor, on both cerebrovascular pulsatility and reactivity in patients with cSVD.^6^

Endothelial function can be assessed through CO_2_ inhalation during MRI or transcranial ultrasound, but this is poorly tolerated by many patients and technically challenging. It is also uncertain whether this is principally a marker of established disease or a component of the causative pathway leading to worse cSVD, and therefore a target for treatment. To assess this in clinical populations and future trials, a more acceptable marker of endothelial dysfunction in cSVD is required, ideally one that is already available in very large population-based cohorts.

Neurovascular coupling (NVC) measures the change in cerebral blood flow (CBF) due to neural activity.^7^ It is altered in degenerative conditions like Alzheimer’s,^8,9^ vascular dementia^9^ and Parkinson’s.^10^ The neurovascular unit (NVU) triggers vasodilation in response to neural activity to increase blood flow to meet the metabolic demands of active neurons (NVC).^11^ Vasodilatation is at least partially dependent upon endothelial factors, and therefore NVC reflects endothelial dysfunction.^7^ Furthermore, impaired NVC may therefore be associated with a reduced capacity of the cerebrovascular to respond to haemodynamic challenges, resulting in decreased oxygen and nutrient delivery to the brain, contributing to further neuronal dysfunction and potentially exacerbating cSVD progression.^7^ As such, NVC has the potential to provide a measure of endothelial dysfunction both as a marker of disease severity and as a possible targetable mechanism for treatment.

NVC can be assessed through non-invasive and well-tolerated methods, including blood-oxygen-level-dependent functional MRI (BOLD-fMRI),^12^ arterial spin labelling MR perfusion (ASL),^13^ near-infrared spectroscopy (NIRS),^14^ Oxygen-15-Water Positron Emission Tomography (PET),^15^ single-photon emission computerized tomography (SPECT)^16^ and transcranial Doppler (TCD) ultrasound.^17^ NVC may also therefore provide an alternative measure of endothelial function in cSVD that is available in large population-based studies and easily applicable during clinical MRI imaging.

To evaluate the potential of neurovascular coupling as a reliable marker of cerebrovascular dysfunction, we conducted a systematic review and meta-analysis on the reported associations between neurovascular coupling and cSVD severity.

## Material and methods

### Eligibility criteria and search strategy

This systematic review and meta-analysis was registered on PROSPERO (CRD42022382637) and was reported in line with the Preferred Reporting Items for Systematic Reviews and Meta-Analyses (PRISMA) checklist (Supplemental Table 1).^18^ The PubMed and EMBASE databases were searched for potential studies from inception to November 3rd, 2022. Search strategy is detailed in Supplemental List 1. No restriction on species, languages, and study types were applied in initial search. Both authors performed searching and screening of studies. Disagreements were resolved by discussion between two authors. Reference lists were searched for eligible studies.

### Selection of studies

#### Inclusion criteria

(1) Confirmation of cSVD neuroimaging markers on CT or MRI, including WMH, CMB, EPVS or lacunes according to the STRIVE standards^2^ (Supplemental Table 2); (2) Quantified measurements of cSVD neuroimaging markers; (3) Quantified measurements of NVC with BOLD-fMRI, ASL-fMRI, TCD, NIRS, PET or SPECT; (4) Reported relationship between a measure of NVC to a standardized stimulus and severity of a marker of cSVD.

#### Exclusion criteria

(1) No measurements of cSVD neuroimaging markers; (2) No measurements of NVC; (3) No means of neuronal activation (i.e. visual stimulus, motor tasks), including resting-state only studies; (5) Non-human participants; (6) Non-English; (7) Less than 10 participants in a single study; (8) No available full text, including conference abstracts or posters.

### Data extraction and quality assessment

The following data was extracted from the included studies: first author, year of publication, study design, sample size, age, sex, cSVD markers, cSVD markers quantification, methods of neuronal stimulus, methods and outcomes for measuring NVC and direction of effects. If there was inadequate or unclear data, the corresponding authors of the studies were contacted for relevant information or clarification. If the study measured NVC at different timepoints without providing a single quantification across timepoints, changes with greater statistical significance were extracted. The Newcastle-Ottawa Quality Assessment Scale (NOS) was used to assess the quality of the included studies.^19^

### Statistical analysis

Studies reporting quantification of the burden of WMH, CMB or lacunes and NVC measurements were assessed for eligibility to be included in the meta-analysis. If a cSVD marker or a NVC assessment location was only reported in one study, they would not be included in the meta-analyses. Furthermore, only studies with consistent tasks and consistent regions of interest could be combined.

Since multiple cSVD markers and NVC in different brain locations or cerebral vessels were evaluated, we grouped the extracted studies by types of cSVD (WMH, CMB, lacunes) and location where NVC was assessed.

Statistical analysis was carried in R (version 4.2.2 with the following packages: database management by *data.table* (version 1.14.8); meta-analysis and plots by *meta* (version 6.2-0). Minimum number of studies for inclusion in meta-analysis is 3. Pool effects were calculated by standardized mean difference (SMD) using the inverse variance (IV) method. Due to high heterogeneity from preliminary analysis, random effects were used. Subgroup analysis based on cSVD aetiology was performed.

The inconsistency test (*I*^2^) was used to assess statistical heterogeneity across included studies, where *I*^2^ values of 25%, 50% and 75% are considered low, medium and high heterogeneity, respectively.

If there were more than 10 studies included in meta-analysis, funnel plots were used to assess publication bias.^20^ For meta-analyses that included 10 or more studies, Egger’s regression test was calculated. A *p*-value of <0.05 was interpreted as statistically significant.

## Results

### Study selection

**Figure 1.**
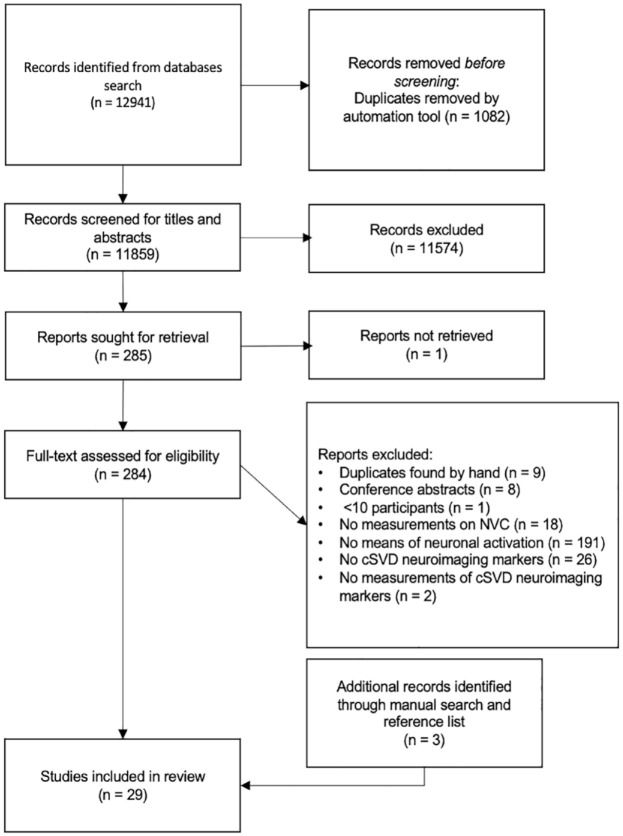
PRISMA flowchart of literature search and selection.

The search yielded 12,941 results. After removal of duplicates and title/abstract screening, 284 studies were retrieved for full-text review. They were evaluated by two reviewers independently, and the reference lists of the included papers were also screened for eligible studies (Figure 1).

### Study characteristics

Twenty-nine studies were included in this systemic review, comprising 19 case-control studies and 10 cohort studies. The characteristics of the included studies are summarized in Table 1 and detailed in Supplemental Table 3. The mean age in the higher-cSVD burden group, which refers to the group with presence or higher counts/volumes of cSVD markers, was higher at 64.44 compared to 59.54. All included studies assessed cSVD using MRI, with field strength ranging from 1.5 to 7 Tesla. WMH was the commonest cSVD marker reported whilst associations between NVC and other markers were less commonly reported, with nine studies reporting associations with CMB, five with lacunes and none with EPVS (Table 2).

**Table 1.** Characteristics of the included studies.

	Total	Higher cSVD burden	Lower cSVD burden
Age, mean (SD)	64.06 (8.16), [28]	64.44 (8.16), [20]	59.54 (8.76), [20]
Sex, female	704/1408 (50%), [29]	297/532 (55.8%), [21]	235/532 (44.2%), [21]
Subjects size	1116, [22]		
WMH	997, [20]	469 (47%)	528 (53%)
CMB	515, [10]	145 (28.2%)	370 (71.8%)
Lacunes	167, [4]	50 (29.9%)	117 (70.1%)

Numbers presented are study size, with study reference.

**Table 2.** NVC changes and cSVD quantification methods.

		Statistically-significant reduced NVC	No statistically-significant change or mixed results	Statistically-significant increased NVC
WMH (*n* = 28)	Presence	*n* = 6	*n* = 1	-
	Number	*n* = 1	*n* = 4	*n* = 2
	Volume	*n* = 13	*n* = 6	*n* = 1
CMB (*n* = 9)	Presence	*n* = 3	*n* = 1	*n* = 1
	Number	*n* = 5	*n* = 3	-
Lacunes (*n* = 5)	Presence	*n* = 3	*n* = 1	-
	Number	*n* = 1	*n* = 1	-
Total no. of studies	18	13	4	

The majority of studies (22/29) used BOLD-fMRI to measure changes in BOLD signals in respective brain regions. Among two studies combining ASL-fMRI and BOLD-fMRI to detect changes in CBF and BOLD signal, Huneau et al. compared changes in the primary motor and visual cortices in cerebral autosomal dominant arteriopathy with subcortical infarcts and leukoencephalopathy (CADASIL) patients, while Opstal et al. looked at changes in the occipital lobe in hereditary cerebral amyloid angiopathy (CAA) gene carriers. Two studies used NIRS to measure changes in the concentrations of haemoglobin to derive CBF data, with Tak et al. combining BOLD-fMRI with NIRS to look at NVC changes at the primary motor and somatosensory cortices. Five studies used TCD and one used PET (Oxygen-15).

Multiple methods of neuronal stimulus to activate NVC were used. The commonest method was visual stimulation with 12 studies employing a flickering checkerboard-like pattern (8 or 10 Hz) and one by reading a magazine. Motor stimulus by hand or finger movements were performed in six studies and by ankle movement in 1. Cognitive stimulation with an established fMRI paradigm was used in nine studies: the ‘N-back’ task was performed in three studies to activate working memory regions; two studies used the Stroop test to excite regions involving cognitive flexibility and attentional control, whilst other methods to stimulate regions involved in cognitive functions included episodic memory retrieval, verbal memory encoding, sample-matching working memory, Go/No-go and the Digit Symbol Substitution Test. Among these, eight reported correlations between cSVD severity and task performance. Overall, similar task performance was observed in group with higher cSVD burden compared to healthy controls or lower cSVD burden (Supplemental Table 4). Less commonly used neuronal stimuli focused on brain regions involved in affection. Aizenstein et al.^21^ elicited affective reactivity through face-matching and shape-matching, and Vasudev et al. by words with positive, neutral or negative affections.

Out of the 26 (*p* < 0.001)^22^ studies reporting a positive association between increasingly severe cSVD and reduced NVC, 18 were individually significant. Similarly, in 28 studies reporting associations with increasing WMH, 25 reported reduced NVC (19 showing a significant effect). Other markers of cSVD were associated with reduced NVC in: eight of nine studies with CMB (six showing significance); and three of five studies with lacunes. However, no studies reported an association with EPVS (Table 2).

CAA was the most-studied phenotypic cSVD subgroup, among which six out of seven studies reported reduced NVC with more severe disease on MRI and one study reporting no such correlation. Monogenic CADASIL was the most studied genetic cSVD subgroup, and all four in CADASIL reported reduced NVC with increased cSVD severity.

### Quality assessment

The NOS of the included studies ranged from five to eight, with a median of 7 (Supplemental Table 3).

Due to the small numbers of studies included, especially with a consistent test of NVC in a consistent population, it was not possible to reliably assess publication bias through funnel plots, although it remains possible that there was bias due to unpublished studies demonstrating no association between NVC and cSVD severity.

### Associations between NVC and markers of cSVD

#### White matter hyperintensity

NVC was reported to be reduced in patients with WMH versus controls (17 of 18 studies, *p* < 0.001) and in patients with more severe versus less severe WMH (9 of 11 studies). This association was reported across different cognitive tasks (8 of 10), motor tasks (7 of 8) and visual tasks (12 of 12), with responses in corresponding brain regions. Fourteen of 17 studies reported this association among sporadic SVD.

**Figure 2.**
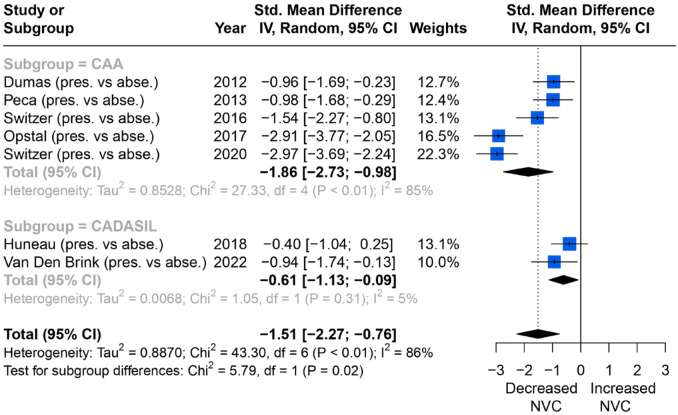
Comparisons of BOLD signal changes in amplitude (%) from baseline in the primary visual cortex between higher and lower WMH burden group.

Seven studies reported a consistent outcome measure of change in percentage of BOLD amplitude in the primary visual cortex with WMH severity (Figure 2), during a flashing checkerboard task, and could therefore be meta-analysed. Studies found that NVC was more impaired in groups with higher WMH burden, with a significant effect on average in a random-effects meta-analysis (SMD = −1.51; 95% CI = −2.27 to −0.76, *p* < 0.01). In subgroup analysis, both CADASIL and CAA patients showed the same association. However, the heterogeneity between studies was high (*I*^2^ = 86%), likely due to a greater effect in patients with CAA. There were no studies reporting a quantitative result in patients with sporadic cSVD.

**Figure 3.**
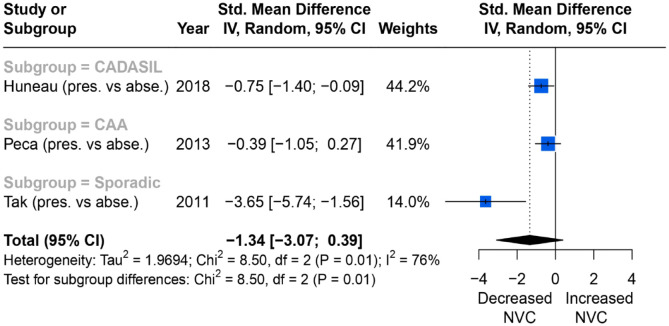
Comparisons of BOLD signal changes in amplitude (%) from baseline in the primary motor cortex between higher and lower WMH burden group.

Overall, six of seven studies reported a reduced BOLD amplitude response in primary motor cortex during a motor task. Of these, three reported consistent associations between change in percentage BOLD amplitude in the primary motor cortex with WMH severity during a motor task, and could be meta-analysed. Overall, impaired NVC was associated with higher WMH burden (SMD = −1.34; 95% CI = −3.07 to 0.39, *p* = 0.01), although there was significant between-studies heterogeneity (*I*^2^ = 76%) (Figure 3).

**Figure 4.**
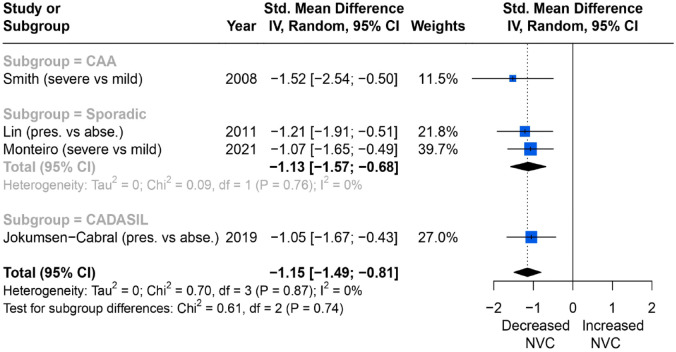
Comparisons of blood flow velocity changes (%) measured by TCD in the PCA (posterior circulation) between higher and lower WMH burden group.

Severity of WMH and NVC assessed on posterior cerebral artery (PCA) TCD to visual stimulus were reported in four studies. All four showed that more WMH were associated with more impaired NVC but the results were not statistically significant (SMD = −1.15; 95% CI = −1.49 to −0.81, *p* = 0.87), with low heterogeneity between studies (*I*^2^ = 0%) (Figure 4).

#### Cerebral microbleeds

**Figure 5.**
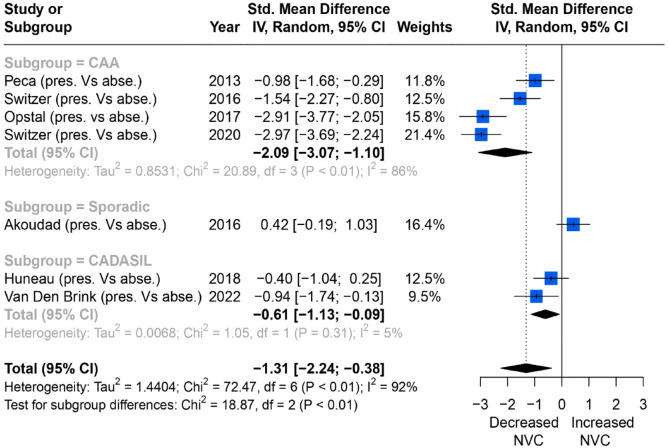
Comparisons of BOLD signal changes in amplitude (%) from baseline in the primary visual cortex between higher and lower CMB burden group.

Of nine studies on CMB, seven reported consistent quantitative associations between the severity of CMB and NVC measured by changes in percentage of BOLD amplitude in the primary visual cortex. Overall, higher CMB burden was associated with more impaired NVC (SMD = −1.31; 95% CI = −2.24 to −0.38, *p* < 0.01), but the heterogeneity was high (*I*^2^ = 92%). In subgroup analysis, CAA and CADASIL both showed the same direction of effects compared to the overall trend, although the single study reporting an association in sporadic cSVD showed a trend in the opposite direction (Figure 5).

#### Lacunes

Two case-control studies reported on the severity of lacunes and NVC measured by changes in percentage of BOLD amplitude in the primary visual cortex. Both recruited CADASIL patients and healthy comparisons, and showed higher lacune burden with worse NVC.

## Discussion

Across 29 studies, there was a highly consistent association between markers of cSVD and reduced NVC, including in meta-analyses of 13 studies with reasonably consistent methods of measurement and analysis. This association was most commonly reported in patients with increased WMH, but was consistent for all markers of sporadic cSVD where there was data available, and was largely consistent for both sporadic disease and for specific cSVD (CADASIL, CAA), implying a common association with endothelial dysfunction. Furthermore, where meta-analysis was possible, there was a consistently reduced response to visual stimuli with all markers of cSVD, despite preservation of vision in these populations, supporting a probable vascular rather than neuronal mechanism for reduced NVC.

Our findings were consistent with other studies investigating the relationship between cSVD and endothelial dysfunction with different methodologies. Systematic endothelial dysfunction is found in cSVD patients,^11^ particularly in studies demonstrating reduced flow-mediated vasodilation of the brachial artery with increasing cSVD severity.^23,24^ Similarly, in the brain, the haemodynamic response to a visual stimulus on BOLD was prolonged in hereditary and sporadic CAA versus healthy controls, and was correlated with increased cerebral atrophy in CAA patients.^25,26^ A similarly slowed response to neuronal stimulation was found in probable CAA patients compared to healthy controls with increasing CMB counts.^27^ Quantitative cerebrovascular reactivity using inhaled CO_2_ is the commonest method of assessing cerebrovascular endothelial dysfunction,^28^ with reduced CBF responses in patients with cSVD.^29^ Lacunes and WMH are also associated with increased blood-brain barrier leakage, and with broader blood-based biomarkers of endothelial dysfunction.^30^ Furthermore, decreased BOLD signal responses in the occipital region upon visual stimuli were associated with more severe WMH, CMB and lacunes, in both sporadic and genetic cSVD. Despite the severity of their conditions, participants remained visually intact, suggesting not only common endothelial involvement in all forms of the disease but a likely common vascular cause for the reduced NVC underlying condition rather than localized neuronal damage. These direct measures of endothelial dysfunction in cSVD are consistent with the direction of impairment in NVC in this review, supporting a common underlying vascular mechanism.

The specificity of NVC to cSVD is currently unclear and warrants further research, and may be present in other conditions. However, if impaired NVC is an early cSVD marker, preceding other structural markers (WMH, lacunes and microbleeds), it may enable timely intervention to prevent disease progression and provide a short-term measure of the effect of future treatments in clinical trials. NVC also reveals real-time, functional consequences of vascular damage, aiding comprehensive understanding of cSVD pathology. If NVC is established as a reliable measure of endothelial dysfunction in cSVD, it would offer a method for assessing the clinical determinants of endothelial dysfunction, its prognostic value in large populations, and to investigate the underlying pathophysiological process, given our more detailed cellular understanding of the physiological basis of NVC.^31^

This study has several limitations. First, multiple sources of heterogeneity, such as varying MRI field strengths and NVC acquisition methods across studies, limited the ability to quantitatively compare and standardize results. In particular, variability in neuronal stimulus frequency with limited signal-to-noise ratio may lead to potential underestimation of associations, but this would lead to a conservative underestimation of the overall effect size. Second, the small, selective study populations limits generalizability of the results, reflected in the heterogeneity in the cSVD manifestations. However, subgroup analysis showed a stronger association between higher cSVD severity and impaired NVC in more defined populations. Third, possible publication bias from unpublished data may exist. Most included studies reported decreased NVC with increased cSVD severity, while only four reported opposite direction of effects, although this may simply represent the reliability of the pathogenic mechanism. Fourth, lack of numerical data in the included studies prevented a comprehensive meta-analysis. Finally, more studies are needed to compare NVC impairment in sporadic cSVD with specific cSVD like CAA or CADASIL, which often present with more severe phenotypes.

Future research with larger populations is needed to establish a stronger association between cSVD and impaired NVC, especially in sporadic cases and to determine the direction of causality. Large datasets like UK Biobank, with over 500,000 participants and 50,000 functional brain images, may provide adequate data. Further studies should stratify cSVD subgroups and adopt consistent methods for assessing NVC across different brain regions and neuronal stimuli. The potential advantage of NVC measurements, such as minimal invasiveness, easy setup, real-time recording and independence of patient compliance (e.g. visual flashing checkerboard) imply great potential for research use and clinical practice, if predictive of disease progression or treatment response on an individual level.

In conclusion, this systemic review and meta-analysis demonstrates that NVC is more impaired in patients with more severe cSVD markers, including WMH, CMB and lacunes. WMH, the most-studied marker, is associated with decreased BOLD changes in visual and motor cortices, as well as poorer cerebral flow velocity response in PCA. These findings suggest a central role of endothelial dysfunction in cSVD. Impaired NVC could serve as a potential biomarker for treatment trials and a practical method to assess physiological dysfunction in cSVD if it predicts disease progression or treatment response.

## Supplemental Material

sj-docx-1-eso-10.1177_23969873231196981 – Supplemental material for Associations between neurovascular coupling and cerebral small vessel disease: A systematic review and meta-analysis Click here for additional data file. Supplemental material, sj-docx-1-eso-10.1177_23969873231196981 for Associations between neurovascular coupling and cerebral small vessel disease: A systematic review and meta-analysis by Sheng Yang and Alastair John Stewart Webb in European Stroke Journal
