# Supplementary material for: Associations between neurovascular coupling and cerebral small vessel disease: A systematic review and meta-analysis
Source: Eur Stroke J. 2023 Sep 11;8(4):895–903. doi: 10.1177/23969873231196981 (PMC10683738; doi:10.1177/23969873231196981)
Supplement: sj-docx-1-eso-10.1177_23969873231196981 – Supplemental material for Associations between neurovascular coupling and cerebral small vessel disease: A systematic review and meta-analysis [file sj-docx-1-eso-10.1177_23969873231196981.docx]

**Supplementary Materials Legend**

**Supplementary List 1**. Search strategy.

**Supplementary Table 1**. PRISMA checklist.

**Supplementary Table 2**. Criteria of cerebral small vessel disease neuroimaging markers according to the STRIVE standards.

**Supplementary Table 3**. Characteristics of included studies.

**Supplementary Table 4**. Studies reporting correlation between severity of cSVD and performance on cognitive tasks.

**Supplementary List 1: Search strategy**

EMBASE (OvidSp)

1 exp neurovascular coupling/

2 exp functional neuroimaging/

3 exp transcranial doppler ultrasonography/

4 exp near infrared spectroscopy/

5 exp arterial spin labeling/

6 exp bold signal/

7 exp endothelial dysfunction/

8 neurovascular unit*.mp.

9 neurovascular coupling*.mp.

10 neurovascular function*.mp.

11 neurovascular dysfunction*.mp.

12 cerebral blood flow*.mp.

13 hemodynamic response*.mp.

14 haemodynamic response*.mp.

15 hemodynamic response function*.mp.

16 haemodynamic response function*.mp.

17 endothelial function*.mp.

18 endothelium function*.mp.

19 endothelium damage*.mp.

20 endothelial damage*.mp.

21 endothelium injur*.mp.

22 endothelial injur*.mp.

23 functional magnetic resonance imaging*.mp.

24 fMRI.mp.

25 blood oxygenation level dependent*.mp.

26 BOLD.mp.

27 (transcranial doppler ultrasound* or transcranial doppler*).mp.

28 transcranial doppler sonograph*.mp.

29 transcranial ultrasound*.mp.

30 transcranial sonograph*.mp.

31 TCD.mp.

32 arterial spin labeling*.mp.

33 ASL.mp.

34 near-infrared spectroscopy*.mp.

35 NIRS.mp.

36 exp single photon emission computed tomography/

37 Single Photon Emission Computerized Tomograph*.mp.

38 Single-Photon Emission Computerized Tomograph*.mp.

39 Single Photon Emission CT*.mp.

40 Single-Photon Emission CT*.mp.

41 exp positron emission tomography/

42 SPECT.mp.

43 PET.mp.

44 positron emission tomograph*.mp.

45 1 or 2 or 3 or 4 or 5 or 6 or 7 or 8 or 9 or 10 or 11 or 12 or 13 or 14 or 15 or 16 or 17 or 18 or 19 or 20 or 21 or 22 or 23 or 24 or 25 or 26 or 27 or 28 or 29 or 30 or 31 or 32 or 33 or 34 or 35 or 36 or 37 or 38 or 39 or 40 or 41 or 42 or 43 or 44

1 exp leukoaraiosis/

2 exp perivascular space/

3 exp glymphatic system/

4 exp lacunar stroke/

5 exp CADASIL/

6 exp Binswanger encephalopathy/

7 exp leukoencephalopathy/

8 cerebral microbleed*.mp.

9 cerebral small vessel disease*.mp.

10 Small vessel cerebrovascular disease*.mp.

11 Small vessel cerebrovascular disorder*.mp.

12 Small vessel disease*.mp.

13 SVD.mp.

14 white matter hyperintensiti*.mp.

15 leukoaraiosis*.mp.

16 white matter lesion*.mp.

17 WML.mp.

18 WMH.mp.

19 leucoaraiosis.mp.

20 ischemic leukoaraiosis*.mp.

21 ischaemic leukoaraiosis*.mp.

22 subcortical leukoaraiosis*.mp.

23 white matter change*.mp.

24 WMC.mp.

25 changes in white matter.mp.

26 leukoencephalopathy.mp.

27 white matter disease*.mp.

28 white matter damage*.mp.

29 Binswanger disease*.mp.

30 cerebral white matter disease of Binswanger disease*.mp.

31 microbleed*.mp.

32 cerebral microhemorrhage*.mp.

33 cerebral microhaemorrhage*.mp.

34 dot-like hemosiderin spot*.mp.

35 dot-like hemosiderin deposition*.mp.

36 perivascular space*.mp.

37 PVS.mp.

38 Virchow-Robin space*.mp.

39 etat crible.mp.

40 glymphatic system*.mp.

41 lacun*.mp.

42 lacunar stroke*.mp.

43 recent small subcortical infarct*.mp.

44 CADASIL.mp.

45 "Cerebral Autosomal Dominant Arteriopathy with Sub-cortical Infarcts and Leukoencephalopathy".mp.

46 Cerebral Microangiopath*.mp.

47 subcortical infarct*.mp.

48 brain atroph*.mp.

49 Fazekas scale*.mp.

50 Fazekas.mp.

51 small vessel disease score*.mp.

52 svd score*.mp.

53 small vessel disease burden*.mp.

54 Svd burden*.mp.

55 brain intensity abnormality classification algorithm*.mp.

56 BIANCA.mp.

57 1 or 2 or 3 or 4 or 5 or 6 or 7 or 8 or 9 or 10 or 11 or 12 or 13 or 14 or 15 or 16 or 17 or 18 or 19 or 20 or 21 or 22 or 23 or 24 or 25 or 26 or 27 or 28 or 29 or 30 or 31 or 32 or 33 or 34 or 35 or 36 or 37 or 38 or 39 or 40 or 41 or 42 or 43 or 44 or 46 or 46 or 47 or 48 or 49 or 50 or 51 or 52 or 53 or 54 or 55 or 56

58 45 AND 57

PubMed

#1 "Neurovascular Coupling"[Mesh] OR "Functional Neuroimaging"[Mesh] OR "Ultrasonography, Doppler, Transcranial"[Mesh], "Spectroscopy, Near-Infrared"[Mesh] OR "neurovascular unit*"[tw] OR "neurovascular coupling*" [tw] OR "neurovascular function*" [tw] OR "neurovascular dysfunction*"[tw] OR "cerebral blood flow*"[tw] OR "hemodynamic response*"[tw] OR "haemodynamic response*"[tw] OR "hemodynamic response function*"[tw] OR "haemodynamic response function*"[tw] OR "endothelial function*"[tw] OR "endothelium function*"[tw] OR "endothelium damage*"[tw] OR "endothelial damage*"[tw] OR "endothelium injur*"[tw] OR "endothelial injur*"[tw] OR "functional magnetic resonance imaging*"[tw] OR fMRI[tw] OR "blood oxygenation level dependent*"[tw] OR BOLD[tw] OR "transcranial doppler ultrasound*"[tw] OR "transcranial doppler*"[tw] OR "transcranial doppler sonograph*"[tw] OR "transcranial ultrasound*"[tw] OR "transcranial sonograph*"[tw] OR TCD[tw] OR "arterial spin labeling*"[tw] OR ASL[tw] OR "near-infrared spectroscopy*"[tw] OR NIRS[tw] OR "Positron Emission Tomography Computed Tomography"[Mesh] OR "Tomography, Emission-Computed, Single-Photon"[Mesh] OR "positrion emission tomography computed tomograph*"[tw] OR PET[tw] OR "Single-Photon Emission Computerized Tomograph*"[tw] OR "Single Photon Emission Computerized Tomograph*"[tw] OR SPECT[tw] OR "Single-Photon Emission CT*"[tw] OR "Single Photon Emission Computerized Tomograph*"[tw]

#2 "Cerebral Small Vessel Disease*"[Mesh] OR "Leukoaraiosis"[Mesh] OR "perivascular space"[Mesh] OR "Glymphatic System"[Mesh] OR "Stroke, Lacunar"[Mesh] OR "CADASIL"[Mesh] OR "cerebral small vessel disease*"[tw] OR "Small vessel cerebrovascular disease*"[tw] OR "Small vessel cerebrovascular disorder*"[tw] OR "Small vessel disease"[tw] OR SVD[tw] OR "white matter hyperintensiti*"[tw] OR "white matter lesion*"[tw] OR WML[tw] OR WMH[tw] OR leukoaraiosis*[tw] OR leucoaraiosis*[tw] OR "ischemic leukoaraiosis*"[tw] OR "ischaemic leukoaraiosis*"[tw] OR "subcortical leukoaraiosis*"[tw] OR "white matter change*"[tw] OR WMC[tw] OR "changes in white matter"[tw] OR "leukoencephalopathy"[tw] OR "white matter disease*"[tw] OR "white matter damage*"[tw] OR "Binswanger’s disease*"[tw] OR "cerebral white matter disease of Binswanger’s disease*"[tw] OR "cerebral microbleed*"[tw] OR "microbleed*"[tw] OR "cerebral microhemorrhage*"[tw] OR "cerebral microhaemorrhage*"[tw] OR "dot-like hemosiderin spot*"[tw] OR "dot-like hemosiderin deposition*"[tw] OR "perivascular space*"[tw] OR PVS[tw] OR "Virchow-Robin space*"[tw] OR "etat crible"[tw] OR "glymphatic system*"[tw] OR lacun*[tw] OR "lacunar stroke*"[tw] OR "recent small subcortical infarct*"[tw] OR CADASIL[tw] OR "Cerebral Autosomal Dominant Arteriopathy with Sub-cortical Infarcts and Leukoencephalopathy"[tw] OR "Cerebral Microangiopath*"[tw] OR "subcortical infarct*"[tw] OR "brain atroph*"[tw] OR "Fazekas scale*"[tw] OR Fazekas[tw] OR "small vessel disease score*"[tw] OR "svd score*"[tw] OR "small vessel disease burden*"[tw] OR "svd burden*"[tw] OR "brain intensity abnormality classification algorithm*"[tw] OR BIANCA[tw]

#3 #1 AND #2

English, from inception to Nov 3^rd^, 2022

**Supplementary Table 1. PRISMA checklist.**

| **Section and Topic** | **Item #** | **Checklist item** | **Location where item is reported** |
| --- | --- | --- | --- |
| **TITLE** | | |  |
| Title | 1 | Identify the report as a systematic review. | p. 1 |
| **ABSTRACT** | | |  |
| Abstract | 2 | See the PRISMA 2020 for Abstracts checklist. | p. 2 |
| **INTRODUCTION** | | |  |
| Rationale | 3 | Describe the rationale for the review in the context of existing knowledge. | p. 5 |
| Objectives | 4 | Provide an explicit statement of the objective(s) or question(s) the review addresses. | p. 7 |
| **METHODS** | | |  |
| Eligibility criteria | 5 | Specify the inclusion and exclusion criteria for the review and how studies were grouped for the syntheses. | p. 8-9 |
| Information sources | 6 | Specify all databases, registers, websites, organisations, reference lists and other sources searched or consulted to identify studies. Specify the date when each source was last searched or consulted. | p. 8 |
| Search strategy | 7 | Present the full search strategies for all databases, registers and websites, including any filters and limits used. | Supplementary List 1 |
| Selection process | 8 | Specify the methods used to decide whether a study met the inclusion criteria of the review, including how many reviewers screened each record and each report retrieved, whether they worked independently, and if applicable, details of automation tools used in the process. | p. 8 |
| Data collection process | 9 | Specify the methods used to collect data from reports, including how many reviewers collected data from each report, whether they worked independently, any processes for obtaining or confirming data from study investigators, and if applicable, details of automation tools used in the process. | p. 9 |
| Data items | 10a | List and define all outcomes for which data were sought. Specify whether all results that were compatible with each outcome domain in each study were sought (e.g. for all measures, time points, analyses), and if not, the methods used to decide which results to collect. | p. 8-9 |
|  | 10b | List and define all other variables for which data were sought (e.g. participant and intervention characteristics, funding sources). Describe any assumptions made about any missing or unclear information. | p. 8-9 |
| Study risk of bias assessment | 11 | Specify the methods used to assess risk of bias in the included studies, including details of the tool(s) used, how many reviewers assessed each study and whether they worked independently, and if applicable, details of automation tools used in the process. | p. 8, 9-11 |
| Effect measures | 12 | Specify for each outcome the effect measure(s) (e.g. risk ratio, mean difference) used in the synthesis or presentation of results. | p. 9-10 |
| Synthesis methods | 13a | Describe the processes used to decide which studies were eligible for each synthesis (e.g. tabulating the study intervention characteristics and comparing against the planned groups for each synthesis (item #5)). | p. 9-11 |
|  | 13b | Describe any methods required to prepare the data for presentation or synthesis, such as handling of missing summary statistics, or data conversions. | p. 9-11 |
|  | 13c | Describe any methods used to tabulate or visually display results of individual studies and syntheses. | p. 9-11 |
|  | 13d | Describe any methods used to synthesize results and provide a rationale for the choice(s). If meta-analysis was performed, describe the model(s), method(s) to identify the presence and extent of statistical heterogeneity, and software package(s) used. | p. 9-11 |
|  | 13e | Describe any methods used to explore possible causes of heterogeneity among study results (e.g. subgroup analysis, meta-regression). | p. 9-11 |
|  | 13f | Describe any sensitivity analyses conducted to assess robustness of the synthesized results. | p. 9-11 |
| Reporting bias assessment | 14 | Describe any methods used to assess risk of bias due to missing results in a synthesis (arising from reporting biases). | p. 11 |
| Certainty assessment | 15 | Describe any methods used to assess certainty (or confidence) in the body of evidence for an outcome. | p. 10 |
| **RESULTS** | | |  |
| Study selection | 16a | Describe the results of the search and selection process, from the number of records identified in the search to the number of studies included in the review, ideally using a flow diagram. | Figure 1 |
|  | 16b | Cite studies that might appear to meet the inclusion criteria, but which were excluded, and explain why they were excluded. | Figure 1 |
| Study characteristics | 17 | Cite each included study and present its characteristics. | Table 2 , Supplementary Table 3 |
| Risk of bias in studies | 18 | Present assessments of risk of bias for each included study. | p. 15, Supplementary Table 2 |
| Results of individual studies | 19 | For all outcomes, present, for each study: (a) summary statistics for each group (where appropriate) and (b) an effect estimate and its precision (e.g. confidence/credible interval), ideally using structured tables or plots. | Figure 2-5, Supplementary table 3 |
| Results of syntheses | 20a | For each synthesis, briefly summarise the characteristics and risk of bias among contributing studies. | p. 12-15 |
|  | 20b | Present results of all statistical syntheses conducted. If meta-analysis was done, present for each the summary estimate and its precision (e.g. confidence/credible interval) and measures of statistical heterogeneity. If comparing groups, describe the direction of the effect. | p. 15-18 |
|  | 20c | Present results of all investigations of possible causes of heterogeneity among study results. | p. 15-18 |
|  | 20d | Present results of all sensitivity analyses conducted to assess the robustness of the synthesized results. | p. 15-18 |
| Reporting biases | 21 | Present assessments of risk of bias due to missing results (arising from reporting biases) for each synthesis assessed. | p. 15 |
| Certainty of evidence | 22 | Present assessments of certainty (or confidence) in the body of evidence for each outcome assessed. | p. 12, Supplementary table 3 |
| **DISCUSSION** | | |  |
| Discussion | 23a | Provide a general interpretation of the results in the context of other evidence. | p. 19 |
|  | 23b | Discuss any limitations of the evidence included in the review. | p. 22-23 |
|  | 23c | Discuss any limitations of the review processes used. | p. 22-23 |
|  | 23d | Discuss implications of the results for practice, policy, and future research. | p. 23 |
| **OTHER INFORMATION** | | |  |
| Registration and protocol | 24a | Provide registration information for the review, including register name and registration number, or state that the review was not registered. | p. 8 |
|  | 24b | Indicate where the review protocol can be accessed, or state that a protocol was not prepared. | - |
|  | 24c | Describe and explain any amendments to information provided at registration or in the protocol. | - |
| Support | 25 | Describe sources of financial or non-financial support for the review, and the role of the funders or sponsors in the review. | p. 24 |
| Competing interests | 26 | Declare any competing interests of review authors. | p. 24 |
| Availability of data, code and other materials | 27 | Report which of the following are publicly available and where they can be found: template data collection forms; data extracted from included studies; data used for all analyses; analytic code; any other materials used in the review. | Supplementary table 3 |

**Supplementary Table 2. Criteria of cerebral small vessel disease neuroimaging markers according to the STRIVE standards.^1^**

| cSVD markers | Proposed terms and definitions for neuroimaging features of small vessel disease |
| --- | --- |
| White matter hyperintensity of presumed vascular origin (WMH) | Signal abnormality of variable size in the white matter that shows the following characteristics: hyperintensity on T2-weighted images such as fluid-attenuated inversion recovery, without cavitation (signal different from CSF). Lesions in the subcortical grey matter or brainstem are not included in this category unless explicitly stated. If deep grey matter and brainstem hyperintensities are also included, the collective term should be subcortical hyperintensities. |
| Cerebral microbleed (CMB) | Small (generally 2–5 mm in diameter, but sometimes up to 10 mm) areas of signal void with associated blooming seen on T2*-weighted MRI or other sequences that are sensitive to susceptibility effects. |
| Lacune of presumed vascular origin (Lacune) | A round or ovoid, subcortical, fluid-filled cavity (signal similar to CSF) of between 3 mm and about 15 mm in diameter, consistent with a previous acute small subcortical infarct or haemorrhage in the territory of one perforating arteriole. |
| Perivascular space (EPVS) | Fluid-filled spaces that follow the typical course of a vessel as it goes through grey or white matter. The spaces have signal intensity similar to CSF on all sequences. Because they follow the course of penetrating vessels, they appear linear when imaged parallel to the course of the vessel, and round or ovoid, with a diameter generally smaller than 3 mm, when imaged perpendicular to the course of the vessel. |

**Supplementary Table 3. Characteristics of included studies.**

|  | First author, year | Study design | Subjects and age (mean ± SD) | cSVD markers | Neuronal stimulus | NVC measurements and area | Primary outcome | Results | NOS |
| --- | --- | --- | --- | --- | --- | --- | --- | --- | --- |
| 1 | Huneau 2018^2^ | Case-control | 19 CADASIL patients (43.6 ± 6.7) and 19 HC (43.2 ± 8.0). | In patients:  Prescence of WMH (n=19), lacunes (n=4), CMB (n=1), cerebral atrophy (n=0).  In HC: all was absent of cSVD. | Visual stimulation by flickering checkerboard-like pattern (6 Hz, 20 or 40 secs, 6 sequences) with simultaneous visually-cued motor stimulation by opening-closing hand movements. | 3T MRI. CBF obtained by pCASL with T2*- weighted EPI and BOLD signal measured  simultaneously in primary visual and motor cortex. | Percentage of changes in CBF and BOLD signal from baseline. | Reduced changes of CBF and BOLD signal in patients compared with HC. | 8 |
| 2 | Opstal 2017^3^ | Case-control | 15 HCHWA-D CAA patients with symptoms (55 ± 5), 12 HCHWA-D CAA patients without symptoms (34 ± 12), and 33 HC (46 ± 14). | In HCHWA-D CAA patients with symptoms:  WMH volume (cm^3^): 92.4 (2.1-180.6), microbleeds count: 42 (2-468).  In HCHWA-D CAA patients without symptoms:  WMH volume (cm^3^): 5.8 (0.0-37.3), microbleeds count: 0 (0-32).  In HC:  WMH volume (cm^3^): 2.3 (0.0-23.3), microbleeds count: 0 (0-1). | Visual stimulation by flickering checkerboard-like pattern (8 Hz, 20 secs, 16 blocks). | 3T MRI. BOLD signal measured by gradient-echo EPI in visually-stimulated areas.  Regional CBF (rCBF) obtained by pCASL. | Percentage of changes of BOLD signal from baseline, TTP (sec), and TTB (sec).  Mean rCBF signal changes. | Decreased percentage of changes of BOLD signal from baseline in overall were observed in symptomatic patients compared to HC, and in HCHWA-D carrier.  Increased TTP in symptomatic patients compared to HC.  Increased TTB in HCHWA-D carrier compared to HC. | 8 |
| 3 | Aizenstein 2011^4^ | Cohort | 33 elderly depressed patients and 27 nondepressed elderly comparisons (71.6 ± 7.5). | WMH volume was similar between depressed and nondepressed groups.  Depressed group was divided into high and low WMH group (above or below median normalized WMH volume). | Affective stimulation by selecting alternating face-matching and shape-matching (5 blocks). | 3T MRI. BOLD signal measured by gradient-echo EPI in limbic systems. | Changes in BOLD signal between two tasks. | Greater signal changes in patients compared with comparisons.  Greater signal changes in high WMH volume group compared with low WMH volume in patients. | 7 |
| 4 | Akoudad 2016^5^ | Case-control | 35 lobar microbleeds patients (64.0 ± 5.4) and 15 age-matched HC (63.9 ± 4.3). | In patients: single CMB (n=21), multiple CMB (n=14)  In HC: all was absent of CMB. | Visual stimulation by flickering checkerboard-like pattern (8 Hz, 20 secs, 16 blocks). | 3T MRI. BOLD signal measured by EPI in the occipital lobe. | Changes in BOLD amplitude from baseline and time-to-peak (TTP) responses. | Higher amplitude of responses in patients compared with HC.  No difference in amplitude and TTP between patients with single or multiple microbleeds. | 8 |
| 5 | Brown 2018^6^ | Cohort | 35 elderly adults (76.7 ± 7.2). | Total WMH volume and DMN-WMH, which is calculated as volume of regions of DMN deactivation^7^. | Cognitive stimulation with a delayed-match-to-sample working memory task (8 blocks). | 3T MRI. BOLD signal measured by gradient-echo EPI in regions showing DMN deactivation. | DMN deactivation, calculated as inverted percentage of changes in BOLD signal from baseline (more negative % signal change represented more positive deactivation magnitude). | DMN deactivation magnitude was not correlated with DMN-WMH volume. | 6 |
| 6 | Chiti 2018^8^ | Case-control | 14 vascular MCI (VMCI) patients (75.8 ± 7.2), 21 non-vascular MCI (NVMCI) patients (74.0 ± 13), and 15 HC (71.9 ± 7.9). | VMCI: moderate-to-severe WMH (grade 2 or 3 in modified Fazekas scale^9^).  NVMCI and HC: without or mild WMH (grade 0 or 1 of modified Fazekas scale). | Cognitive stimulation by a verbal memory encoding task (27 blocks).^10^ | 3T MRI. BOLD signal measured by gradient-echo EPI in activated regions. | Changes in BOLD signal and pattern of cortical activation from baseline. | Higher BOLD signal changes in the right parieto-occipital cortex, and reduced changes in the left superior and middle frontal gyri, anterior cingulum and in left frontal-opercular area in VMCI patients compared with HC.  Significantly lower signal changes in left superior and middle frontal gyri, in anterior cingulum and in left frontal-opercular area in VMCI patients compared with NVMCI. | 8 |
| 7 | Dumas 2012^11^ | Case-control | 25 probable CAA patients (Boston Criteria^12^) (70.2 ± 7.8) and 12 HC (75.3 ± 6.2). | Normalized WMH (nWMH) volume and CMB numbers.  HC was absent of cSVD on MRI. | Visual stimulation by flickering checkerboard-like pattern (8 Hz, 20 secs, 16 blocks). | 1.5T MRI. BOLD signal measured by gradient-echo EPI in the occipital lobe. | Percentage of changes in BOLD amplitude from baseline, TTP, time-to-baseline (TTB). | Significantly lower amplitude of response, longer TTP and TTB in patients compared with controls.  Longer TTP was moderately correlated with nWMH volume in patients.  No difference in fMRI parameters was observed with CMB numbers in patients. | 7 |
| 8 | Fernández-Cabello 2016^13^ | Cohort | 90 cognitively normal elderly adults (67 ± 2.90), median YoE=12 years, divided into high YoE (n=51) and low YoE (n=39) groups, and 16 young adults (22 ± 1.93). | Median subcortical WMH volume, divided into high WMH and lower WMH.  High YoE, high WMH (n=30)  High YoE, low WMH (n =21)  Low YoE, high WMH (n =15)  Low YoE, low WMH (n =24) | Cognitive stimulation by N-Back working memory tasks (16 blocks, 26 secs each).^14^ | 3T MRI. BOLD signal measured by EPI in regions of significant activation. | Percentage of changes in BOLD signal from baseline. | High education and high WMH burden group showed greatest activation, while low education with high WMH burden showed the least activation. | 6 |
| 9 | Gavazzi 2019^15^ | Case-control | 21 CADASIL patients (44 ± 12) and 16 HC (38.3 ± 11). | In patients:  Fazekas score 0 (n=5), score 1 (n=0), score 2 (n=3), score 3 (n=4), score 4 (n=3), score 5 (n=4), and score 6 (n=2).  Few (1–3) and multiple (>3) lacunes (n=3).  In HC: all was absent of cSVD. | Cognitive stimulation by a Go/No-go task to activate regions of proactive and reactive inhibition (48 trials).^16^ | 3T MRI. BOLD signal measured by EPI in regions of significant activation. | Percentage of changes in BOLD signal from baseline. | Significantly lower BOLD signal changes in 6 clusters (anterior cingulate cortex, the insula, the thalamus, and the lower brain stem) in patients compared with HC.  No clusters of significantly lower signal changes were observed in patients compared with HC. | 7 |
| 10 | Gokcal 2022^17^ | Cohort | 38 cognitively normal probable CAA patients (70 ± 7.1). | Lacunes (n=12),  CMB (n=9), and pWMH (% of WMH volume over estimated ICV) was calculated | Visual stimulation by flickering checkerboard-like pattern (8 Hz, 20 secs, 4 blocks). | 1.5T MRI. BOLD signal measured by gradient-echo EPI in primary visual cortex. | Changes in BOLD signal TTP (BOLD-TTP) from baseline. | Prolonged BOLD-TTP was correlated with higher pWMH, while no association was observed between BOLD-TTP and the presence of lacunes and/or CMB. | 7 |
| 11 | Linortner 2012^18^ | Cohort | 30 healthy adults (67.8 ± 7.5). | Subjects divided into two groups based on modified Fazekas scale: Grade 0 & 1 (n=17) and Grade 2 & 3 (n=13). | Motor stimulation by active right fingers and right ankle movements (1 Hz for fingers, 1000 ms for ankle, 30 secs per block, total 10.5 minutes). | 3T MRI. BOLD signal measured by gradient-echo EPI in the primary and secondary sensori-motor cortices, supplementary and cingulate motor areas, and the cerebellum. | Percentage of changes of BOLD signal from baseline. | Significantly higher BOLD signal changes were noted in higher WMH grade during ankle movement but not  in finger movements. | 6 |
| 12 | Mascalchi 2014^19^ | Cohort | 60 MCI patients (74.7 ± 7.1). | Subjects divided into two groups based on modified Fazekas scale: mild (grade 1, n=14) and moderate-to-severe (grade 2 or 3, n=46). | Motor stimulation by continuously right hand tapping at a self-paced frequency without visual feedback (6 blocks, 30 secs each). | 1.5T MRI. BOLD signal measured by T2* weighted EPI in areas related to motor tasks. | Percentage of changes in BOLD signal from baseline. | Hyperactivation of left precentral and postcentral gyrus and right cerebellar lobule 5 was observed in moderate-to-severe group compared with mild group.  No area of hypoactivation was observed in the moderate-to-severe group compared with mild group. | 7 |
| 13 | Nordahl 2006^20^ | Cohort | 15 cognitively normal adults (78.7 ± 6.06). | WMH volume at global and dorsal PFC. | Cognitive stimulation by an episodic memory retrieval task (to activate PFC and hippocampus) (6 blocks), a verbal item recognition working memory task (to activate dorsolateral PFC) (4 blocks), and a visual sensory control as control (16 secs).  Total: 6 scans. | 1.5T MRI. BOLD signal measured by gradient-echo EPI in global and dorsal prefrontal cortex. | Percentage of changes in BOLD signal from baseline. | Decreased in PFC activity was observed with increased global and dorsal PFC WMH volume.  Decreased activity in medial temporal and anterior cingulate regions during episodic retrieval and decreased activity in the posterior parietal and anterior cingulate cortex during working memory task were observed with increased dorsal PFC WMH volume. | 7 |
| 14 | Papma 2013^21^ | Case-control | 15 MCI patients with cSVD (74.3 ± 4.4), 26 MCI patients without cSVD (72.8 ± 4.4), and 25 HC (71.6 ± 5.2). | Patients with cSVD:  Presence of severe WMH (Fazekas scale ≥2) affecting both anterior and posterior WM regions, and/or presence of ≥2 lacunes.  Patients without cSVD and HC: all was absent of cSVD. | Cognitive stimulation by visual N-Back working memory tasks (9 blocks). | 3T MRI. BOLD signal measured by gradient-echo EPI in regions activated by task. | Percentage of changes in BOLD signal from baseline. | No difference in brain activation was observed between MCI patients with cSVD and HC and between MCI patients with and without cSVD.  Reduced activation in a DMN region was noted in MCI patients with cSVD. | 7 |
| 15 | Patel 2012^22^ | Cohort | 33 depressed elderly adults (68.3 ± 6.6) and 41 nondepressed elderly adults (71.7 ± 7.9). | Normalized WMH volume (nWMH), calculated by whole-brain WMH volume normalized by whole-brain volume. | Motor stimulation by tapping the right index finger to visual cues (5 blocks, 30 secs each). | 3T MRI. BOLD signal measured by gradient-echo EPI in regions activated by task. | Percentage of changes in BOLD signal from baseline.  Depressed and non-depressed groups had similar nWMH volume so data was pooled. | Reduced BOLD signal change in the parietal white matter was observed in higher WMH burden group. | 7 |
| 16 | Peca 2013^23^ | Case-control | 16 probable CAA patients (72.0 ± 7.0) and 16 HC (69.8 ± 7.0). | In patients:  Mean WMH volume (mL): 27.2 (7.8-50.6), mean CMB count: 5 (5-132).  In HC:  Mean WMH volume (mL): 4.3 (3.1-8.4), mean CMB count: 0 (0-0). | Visual stimulation by flickering checkerboard-like pattern (8 Hz, 40 secs, 4 blocks) and a separate motor stimulation by tapping the dominant hand fingers (1.5 Hz, 40 secs, 4 blocks). | 3T MRI. BOLD signal measured by gradient-echo EPI in primary visual and motor cortex. | Percentage of changes in BOLD signal from baseline. | Reduced BOLD signal changes in visual cortex but not motor cortex were observed in patients compared with HC.  Reduced BOLD signal change in visual cortex was correlated with higher mean WMH volume and more CMB in patients. | 8 |
|  | Williams 2017^24^  *The data in this study is a re-analysis of data from Peca et al. 2017^23^ | Case-control | 13 probable CAA patients (74.3 ± 9.2) and 14 HC (68.4 ± 9.4). | nWMH volume and CMB counts. | Visual stimulation by flickering checkerboard-like pattern (8 Hz, 40 secs, 4 blocks) and a separate motor stimulation by tapping the dominant hand fingers (1.5 Hz, 40 secs, 4 blocks). | 3T MRI. BOLD signal measured by gradient-echo EPI in primary visual and motor cortex. | Changes in BOLD-TTP, full-width at half-maximum (FWHM), and area-under-curve (AUC). | For visual cortex:  Reduced AUC in patients compared to HC, and wider FHWM correlated with higher CMB counts.  For motor cortex:  Delayed TTP and wider FWHM in patients compared to HC. | 7 |
| 17 | Switzer 2016^25^ | Case-control | 22 probable CAA patients (72.6 ± 6.9) and 16 HC (68.4 ± 5.9). | In patients:  Baseline WMH volume (mL): 24.8 (10.77-44.01), WMH volume change (mL): +1.36 (-0.48-7.37), baseline CMB count: 24 (6-54), new CMB at one year: n=11, new CMB count: 1 (0-23).  In HC:  Baseline WMH volume (mL): 2.34 (1.71-5.27), WMH volume change (mL): +0.33 (-0.15-1.09), baseline CMB count: 0, new CMB at one year: n=0, new CMB count: 0. | Visual stimulation by flickering checkerboard-like pattern (8 Hz, 40 secs, 4 blocks). | 3T MRI. BOLD signal measured by gradient-echo EPI in primary visual cortex. | Percentage of changes in BOLD signal from baseline. | Significant reduced BOLD signal change was observed in patients, while no reduction or increase was noted in HC.  Reduction in BOLD signal change was not correlated with baseline WMH volume, WMH volume change, baseline CMB count, or new CMB count. | 8 |
| 18 | Switzer 2020^26^ | Case-control | 40 probable CAA patients (74.2 ± 7.3), 22 AD patients (70.0 ± 8.1), 27 MCI patients (71.6 ± 6.7), and 25 HC (67.7 ± 9.4). | In CAA patients:  WMH volume (mL): 32.5 (10.6-46.6), total CMB count: 22 (6-58).  In AD patients:  WMH volume (mL): 8.0 (4.6-11.1), total CMB count: 0 (0-0).  In MCI patients:  WMH volume (mL): 4.2 (2.1-7.4), total CMB count: 0 (0-0).  In HC:  WMH volume (mL): 2.4 (1.5-4.8), total CMB count: 0 (0-0). | Visual stimulation by flickering checkerboard-like pattern (8 Hz, 40 secs, 4 blocks) . | 3T MRI. BOLD signal measured by gradient-echo EPI in primary visual cortex. | Percentage of changes in BOLD signal from baseline. | Reduced BOLD signal change was observed in CAA patients compared with HC, while this reduction was not observed in AD patients and MCI patients compared with HC.  Reduced BOLD signal change was correlated with higher WMH volume in CAA patients, while this correlation was not noted in AD patients, MCI patients, and HC. | 8 |
| 19 | Van Den Brink 2022^27^ | Case-control | 23 CADASIL patients (51.1 ± 10.1) and 13 HC (46.1 ± 12.6). | In CADASIL patients:  *WMH (%): 3.87 (2.06-5.56), presence of lacunes: n=13, lacunes count: 4 (3-8), presence of CMB: n=13, CMB count: 3 (2-8).  In HC:  WMH (%): 0.01 (0.00-0.03), presence of lacunes: n=0, lacunes count: 0 (0-0), presence of CMB: n=0, CMB count: 0 (0-0).  *WMH is presented as % of ICV. | Visual stimulation by flickering checkerboard-like pattern (8 Hz, 16.72 secs, 3 blocks). | 7T MRI. BOLD signal measured by gradient-echo EPI in primary visual cortex. | Percentage of changes in BOLD signal from baseline. | Reduced BOLD signal change was observed in CADASIL patients compared to HC. | 8 |
| 20 | Vasudev 2018^28^ | Case-control | 15 elderly patients with major depressive disorder (Male 68.4 ± 6.8, Female 66.4 ± 5.7) and 14 elderly HC (Male 70.3 ± 4.5, Female 61.9 ± 5.0). | In patients:  WMH volume (mL): 7.2 ± 6.8.  In HC:  WMH volume (mL): 5.1 ± 10.6.  WMH volume, normalized by divided by total brain volume. | Affective stimulation by viewing positive, neutral, and negative affective words, and pressed buttons accordingly (4 runs). | 3T MRI. BOLD signal measured by EPI in regions activated by task. | Percentage of changes in BOLD signal from baseline. | No difference in BOLD signal change was observed in patients compared to HC. | 7 |
| 21 | Venkatraman 2009^29^ | Cohort | 15 elderly adults (81.67 ± 3.47). | nWMH volume and atrophy index calculated as the ratio of CSF volume and gray matter volume. | Cognitive stimulation by The Digit Symbol Substitution Test (10 blocks)^30^ | 3T MRI. BOLD signal measured by gradient-echo EPI in regions activated by the task | Percentage of changes in BOLD signal from baseline. | BOLD signal change in bilateral activated regions was negatively correlated with nWMH volume but not with atrophy index. | 7 |
| 22 | Schroeter 2007^31^ | Case-control | 12 cerebral microangiopathy patients (61.2 ± 4.9) and 12 HC (64.5 ± 2).  MRI was not performed in HC. | Severity of lacunes and  periventricular WMH graded by microangiopathy severity scale^32^, with max scores of 21.  Patients’ mean score: 13 ± 3.3. | Cognitive stimulation by the Stroop Test^33^. Subjects were asked to name the color of the letters while ignoring the meaning of the word (30 trials). | NIRS measured concentration of oxy- and deoxy-Hb at dorsolateral PFC. | Changes in the concentration of oxy- and deoxy-Hb (as CBV) and changes in the concentration of total Hb and Hb difference (as CBF). | Delay and reduced amplitude of the hemodynamic response was observed in patients compared with HC.  However, no significant correlation between hemodynamic response and the severity of CMA in patients. | 6 |
| 23 | Tak 2011^34^ | Case-control | 6 cSVD patients (76.5 ± 3.1) and 6 HC (73.3 ± 1.2). | In patients:  WMH volume (cc): 37.2 ± 6.1.  In HC:  WMH volume (cc): 2.0 ± 0.9. | Motor stimulation by hand grasp to visual “Go” and “Stop” cue (10 tasks, 21 secs each). | NIRS and simultaneous 3T MRI. BOLD signal measured by EPI in primary motor and somatosensory cortices. | Changes in the concentration of oxy- and deoxy-Hb (as CBV),  changes in the concentration of total Hb, and percentage of changes in BOLD signal from baseline. | Significantly decreased oxy-Hb, total Hb, and BOLD changes were observed in patients compared with HC.  This was not observed in deoxy-Hb. | 6 |
| 24 | Videbech 2004^35^ | Case-control | 41 major depression adult patients (41.4 ± 12.5) and 46 HC (41.1 ± 11.6). | In patients:  Subjects with WMH: n=17, mean WMH count: 3.1.  In HC:  Subjects with WMH: n=20, mean WMH count: 2.7.  Overall frequency of WMH and number of subjects did not differ (Fazekas scale grade 1) between two groups. | Cognitive stimulation by the Stroop Test (stimulus displayed for 1 sec). | PET scan with radioactive  water (H_2_^15^O) in areas activated by task. | Regional CBF change. | No difference in CBF was observed in the fronto-striatal white matter and insula in subjects with WMH compared to subjects without WMH. | 7 |
| 25 | Jokumsen-Cabral 2019^36^ | Case-control | 27 CADASIL patients (57 ±13) and 20 HC (59 ± 16). | Age-related white matter change (ARWMC) scale^37^ graded WMC severity (0-3 points) in five regions in each hemisphere (total 30 points). | Visual stimulation by flickering checkerboard-like pattern (10 Hz, 40 secs, 10 cycles). | TCD measured CBF velocity in the M1 segment of the right MCA and the P2 segment of left PCA. | Percentage of PCA CBF velocity change from baseline and MCA response (as negative control). | Reduced CBF velocity change and altered hemodynamics were observed in patients compared with HC.  However, NCV parameters were not correlated with WMH burden (ARWMC) in CADASIL. | 7 |
| 26 | Lin 2011^38^ | Case-control | 21 cSVD patients (64 ± 14), 13 large intracranial artery stenosis patients (LIAS) (58 ± 14), and 17 HC (64 ± 13). | In cSVD patients:  Median modified Fazekas scale: 1.  In LIAS patients and HC:  Median modified Fazekas scale: 0. | Visual stimulation by reading magazine without cue (1 minute, 10 cycles). | TCD measured CBF velocity in the P2 segment of bilateral PCA. | Percentage of CBF velocity change from baseline. | Significantly reduced CBF velocity change was observed in both cSVD and LIAS group compared with HC, but this did not differ between cSVD and LIAS. | 8 |
| 27 | Monteiro 2021^39^ | Case-control | 52 hypertensive patients (64 ± 11) and 17 HC (60 ± 16).  MRI was not performed in 12 patients and all HC. | WMH volume normalized for brain volume (nWMH).  In hypertensive patients:  Divided into higher nWMH volume (> 0.14) and lower (≤ 0.14) groups. | Visual stimulation by flickering checkerboard-like pattern (10 Hz, 40 secs, 10 cycles). | TCD measured CBF velocity in the M1 segment of the right MCA and the P2 segment of the left PCA. | Percentage of PCA CBF velocity change from baseline and MCA response (as negative control). | Among hypertensive patients: NVC parameters did not differ in higher and lower nWMH volume in patients. | 6 |
| 28 | Smith 2008^40^ | Case-control | 11 probable CAA patients (73.5 ± 7.4) and 9 HC (70.9 ± 7.9).  MRI was not performed in HC. | WMH volume normalized for brain volume (nWMH). | Visual stimulation by flickering checkerboard-like pattern (10 Hz, 40 secs, 10 cycles). | TCD measured CBF velocity in the P2 segment of PCA with optimal signal, and M1 segment of the contralateral MCA. | Percentage of PCA CBF velocity change from baseline and MCA response (as negative control). | Reduced peak evoked PCA mean flow velocity was observed in higher nWMH volume in patients. | 6 |
| 29 | Sorond 2013^41^ | Cohort | 24 elderly adults (72.9 ± 5.4). | Normal white matter volume and WMH volume. | Cognitive stimulation by N-Back working memory tasks (1-Back and 2-Back)^42^ and control task. | TCD measured CBF velocity in the MCA. | Mean CBF velocity change between control and N-Back tasks. | Higher mean CBF velocity change was associated with overall lower WMH volume. | 5 |

AD: Alzheimer’s disease; BG: basal ganglia; BOLD: blood oxygenation level-dependent; CAA: cerebral amyloid angiopathy; CADASIL: cerebral autosomal dominant arteriopathy with sub-cortical infarcts and leukoencephalopathy; CBF: cerebral blood flow; CBV: cerebral blood volume; CMB: cerebral microbleeds; CS: centrum semiovale; CSF: cerebrospinal fluid; DMN: default mode network; EPI: echo planar imaging; EPVS: enlarged perivascular spaces; Hb: hemoglobin; HC: healthy controls; HCHWA-D: Hereditary cerebral haemorrhage with amyloidosis-Dutch type; ICV: intracranial volume; MCA: middle cerebral artery; MCI: mild cognitive impairment; NIRS: near-infrared spectroscopy; NVC: neurovascular coupling; pCASL: pseudo-continuous arterial spin labeling; PCA: posterior cerebral artery; PET: positron emission tomography; PFC: prefrontal cortex; SD: standard deviation; secs: seconds; TCD: transcranial doppler; TTB: time-to-baseline; TTP: time-to-peak; WM: white matter; WMH: white matter hyperintensities; YoE: year of education.

**Supplementary Table 4. Studies reporting correlation between severity of cSVD and performance on cognitive tasks.**

|  | First author | cSVD markers | Cognitive Stimulus | Results |
| --- | --- | --- | --- | --- |
| 1 | Chiti^8^ | WMH  VMCI: moderate-to-severe WMH (grade 2 or 3 in modified Fazekas scale^9^).  NVMCI and HC: without or mild WMH (grade 0 or 1 of modified Fazekas scale). | Verbal memory encoding task^10,43^ comprised of two parts. The first included the encoding process, where subjects were presented visually words and indicated corresponding feeling (pleasant or unpleasant) by pressing buttons with hands, and the neutral stimuli, which is similar to the encoding process but the words were neutral inscriptions. The second parts included the retrieval process where subjects had to recall words shown in the encoding process and new words where subjects had to remember new words.  The following performance outcomes were recorded: number of responses (encoding process), reaction times (encoding process and neutral stimuli), percentage of correct responses (neutral stimuli, retrieval process, and new words). | Numbers of response and reaction times did not differ significantly between VMCI, NVMCI, and HC.  Percentage of correct response did not differ in three groups in neural stimuli condition, but reached significance between VMCI and NVMCI and between VMCI and HC in retrieval process. It only reached significance between NVMCI patients and HC in new words. |
| 2 | Fernández-Cabello^13^ | 90 cognitively normal elderly adults, median YoE=12 years, divided into high YoE (n=51) and low YoE (n=39) groups, and 16 young adults. | N-Back working memory tasks^14^, in which subjects were presented visually with a sequence of capital letters, and were instructed to press button according to the numbers of repeated letter (1 back, 2 back, or 3 back) or to letter “X” (0 back). Tasks were performed with different load (0 to 3). Hits, false hits, and reaction times were recorded. | Higher education group: higher WMH burden showed higher activation.  Lower education group: higher WMH burden showed least activations and deactivations. |
| 3 | Gavazzi^15^ | In CADASIL patients:  WMH and lacunes.  In HC: absent of cSVD. | Go/No-go task, in which subjects were presented visually a Go stimulus (letter X) or No-go stimulus (letter A), and requested to press or not press. Response times, errors of omission, and commission were recorded. | No statistically significant differences in task performance (correct responses and response times) were found between CADASIL patients and HC. |
| 4 | Nordahl^20^ | WMH volume at dorsal PFC of cognitive normal adults. | An episodic memory retrieval task where subjects were first presented with 36 objects and instructed to memorize the objects and their colors. After one hour they were put into scanner and presented with the objects, and pressed button “Yes” or “No” to whether the colors matched the originals.  A verbal item recognition working memory task where subjects were shown a set of letters, and with a short delay a letter appeared, and they were instructed to identify if the letter matched any letter in the original set. High load (6 letters in set) 5and low load (4 letters in set) were performed. Mean accuracy w6as recorded. | For both tasks, mean accuracy was not significantly associated with global or dorsal PFC WMH volume. |
| 5 | Papma^21^ | MCI Patients with cSVD:  WMH (Fazekas score ≥2).  MCI Patients without cSVD and HC: absent of cSVD. | N-Back working memory tasks^14^, in which subjects presented visually with a sequence of capital letters, and were instructed to press button according to the numbers of repeated letter (1 back or 2 back) or to letter “X” (0 back). Task performance (hit, correct hits, and false hits) and reaction times were recorded. | Similar tasks performance was observed in MCI patients with/without cSVD and HC. Only in 1 back condition, task performance was worse in MCI without cSVD group. |
| 6 | Venkatraman^29^ | WMH volume and cerebral atrophy (atrophy index) of 15 elderly adults. | The Digit Symbol Substitution Test (DSST) measures psychomotor performance and in current study working memory. Subjects performed the paper and pencil DSST and the computerized version DSST (sDSST), in which subjects were presented visually one number-symbol matching pair as cue, then columns of number-symbol matching pairs. They were instructed to press button “Yes” or “No” if the cue matched one of the columns shown as fast as they can. Reaction times and accuracy were recorded. | Lower score on the paper and pencil DSST was significantly associated with greater WMH volume and atrophy index. Lower longer response times accuracy on the sDSST were also associated with greater WMH volume, but failed to reach statistical significance with atrophy index. |
| 7 | Schroeter^31^ | Severity of lacunes and  periventricular WMH graded by microangiopathy severity scale^32^, with max scores of 21.  Patients’ mean score: 13 ± 3.3. | The Stroop Test^33^ measures cognitive flexibility and attentional control. Subjects were presented visually with two rows of letters, and was instructed to press button “Yes” if the color of upper letters matched the lower color name. The test included neutral trials (upper showing “X”), congruent (letter color matched the letter) and incongruent tests (letter color did not match the letter). Congruent tests were not analyzed. Mean errors rates and interference time, calculated as the reaction times difference between congruent and neutral tests, were recorded. | Patients reacted more slowly (longer interference time) than HC. Mean error rates were significantly higher in both groups in the incongruent test, when compared with the neutral trial. |
| 8 | Videbech^35^ | In depressed patients:  Subjects with WMH: n=17, mean WMH count: 3.1.  In HC:  Subjects with WMH: n=20, mean WMH count: 2.7.  Overall frequency of WMH and number of subjects did not differ between two groups. | The Stroop Test, in which subjects were presented visually with colored letters, and were instructed to name the color of the letter rather than the letter itself as quickly as possible. The test included congruent and incongruent tests. Errors rates and interference time, calculated as the reaction times difference between congruent and incongruent tests, were recorded. | Poorer performance on the Stroop Test were associated with presence of WMH in the following locations: the basal ganglia claustrum and extra-nuclear white matter, in the frontal lobe white matter (near the medial frontal gyrus and the precentral gyrus), and in the white matter near the insula, regardless of subjects being patients or HC. |

CADASIL: cerebral autosomal dominant arteriopathy with sub-cortical infarcts and leukoencephalopathy; cSVD: cerebral small vessel disease; HC: healthy controls; MCI: mild cognitive impairment; PFC: prefrontal cortex; WMH: white matter hyperintensities; YoE: year of education.

**Reference**

1. Wardlaw JM, Smith EE, Biessels GJ, Cordonnier C, Fazekas F, Frayne R, Lindley RI, O'Brien JT, Barkhof F, Benavente OR, et al. Neuroimaging standards for research into small vessel disease and its contribution to ageing and neurodegeneration. *Lancet Neurol*. 2013;12:822-838. doi: 10.1016/S1474-4422(13)70124-8

2. Huneau C, Houot M, Joutel A, Beranger B, Giroux C, Benali H, Chabriat H. Altered dynamics of neurovascular coupling in CADASIL. *Annals of Clinical and Translational Neurology*. 2018;5:788-802. doi: doi:<https://dx.doi.org/10.1002/acn3.574>

3. van Opstal AM, van Rooden S, van Harten T, Ghariq E, Labadie G, Fotiadis P, Gurol ME, Terwindt GM, Wermer MJH, van Buchem MA, et al. Cerebrovascular function in presymptomatic and symptomatic individuals with hereditary cerebral amyloid angiopathy: a case-control study. *Lancet Neurol*. 2017;16:115-122. doi: 10.1016/S1474-4422(16)30346-5

4. Aizenstein HJ, Andreescu C, Edelman KL, Cochran JL, Price J, Butters MA, Karp J, Patel M, Reynolds CF, 3rd. fMRI correlates of white matter hyperintensities in late-life depression. *Am J Psychiatry*. 2011;168:1075-1082. doi: doi:10.1176/appi.ajp.2011.10060853

5. Akoudad S, Gurol ME, Fotiadis P, Koudstaal PJ, Hofman A, Ikram MA, Greenberg SM, Vernooij MW. Cerebral Microbleeds and Cerebrovascular Reactivity in the General Population: The EDAN Study. *J Alzheimers Dis*. 2016;53:497-503. doi: doi:10.3233/jad-151130

6. Brown CA, Jiang Y, Smith CD, Gold BT. Age and Alzheimer's pathology disrupt default mode network functioning via alterations in white matter microstructure but not hyperintensities. *Cortex*. 2018;104:58-74. doi: doi:<https://dx.doi.org/10.1016/j.cortex.2018.04.006>

7. Brown CA, Hakun JG, Zhu Z, Johnson NF, Gold BT. White matter microstructure contributes to age-related declines in task-induced deactivation of the default mode network. *Front Aging Neurosci*. 2015;7:194. doi: 10.3389/fnagi.2015.00194

8. Chiti A, Cecchi P, Pesaresi I, Orl, i G, Giannini N, Gialdini G, Terni E, Tognoni G, Volpi L, et al. Functional magnetic resonance imaging with encoding task in patients with mild cognitive impairment and different severity of leukoaraiosis. *Psychiatry Research - Neuroimaging*. 2018;282:126-131. doi: doi:<https://dx.doi.org/10.1016/j.pscychresns.2018.06.012>

9. Fazekas F, Chawluk JB, Alavi A, Hurtig HI, Zimmerman RA. MR signal abnormalities at 1.5 T in Alzheimer's dementia and normal aging. *AJR Am J Roentgenol*. 1987;149:351-356. doi: 10.2214/ajr.149.2.351

10. Daselaar SM, Rombouts SA, Veltman DJ, Raaijmakers JG, Lazeron RH, Jonker C. Parahippocampal activation during successful recognition of words: a self-paced event-related fMRI study. *Neuroimage*. 2001;13:1113-1120. doi: 10.1006/nimg.2001.0758

11. Dumas A, Dierksen GA, Gurol ME, Halpin A, Martinez-Ramirez S, Schwab K, Rosand J, Viswanathan A, Salat DH, Polimeni JR, et al. Functional magnetic resonance imaging detection of vascular reactivity in cerebral amyloid angiopathy. *Ann Neurol*. 2012;72:76-81. doi: 10.1002/ana.23566

12. Knudsen KA, Rosand J, Karluk D, Greenberg SM. Clinical diagnosis of cerebral amyloid angiopathy: validation of the Boston criteria. *Neurology*. 2001;56:537-539. doi: 10.1212/wnl.56.4.537

13. Fernández-Cabello S, Valls-Pedret C, Schurz M, Vidal-Piñeiro D, Sala-Llonch R, Bargallo N, Ros E, Bartrés-Faz D. White matter hyperintensities and cognitive reserve during a working memory task: a functional magnetic resonance imaging study in cognitively normal older adults. *Neurobiol Aging*. 2016;48:23-33. doi: doi:10.1016/j.neurobiolaging.2016.08.008

14. Sala-Llonch R, Arenaza-Urquijo EM, Valls-Pedret C, Vidal-Pineiro D, Bargallo N, Junque C, Bartres-Faz D. Dynamic functional reorganizations and relationship with working memory performance in healthy aging. *Front Hum Neurosci*. 2012;6:152. doi: 10.3389/fnhum.2012.00152

15. Gavazzi G, Orsolini S, Salvadori E, Bianchi A, Rossi A, Donnini I, Rinnoci V, Pescini F, Diciotti S, Viggiano MP, et al. Functional magnetic resonance imaging of inhibitory control reveals decreased blood oxygen level dependent effect in cerebral autosomal dominant arteriopathy with subcortical infarcts and leukoencephalopathy. *Stroke*. 2019;50:69-75. doi: doi:<https://dx.doi.org/10.1161/STROKEAHA.118.022923>

16. Gavazzi G, Orsolini S, Rossi A, Bianchi A, Bartolini E, Nicolai E, Soricelli A, Aiello M, Diciotti S, Viggiano MP, et al. Alexithymic trait is associated with right IFG and pre-SMA activation in non-emotional response inhibition in healthy subjects. *Neurosci Lett*. 2017;658:150-154. doi: 10.1016/j.neulet.2017.08.031

17. Gokcal E, Horn MJ, Becker JA, Das AS, Schwab K, Biffi A, Rost N, Ros, J, Viswanathan A, et al. Effect of vascular amyloid on white matter disease is mediated by vascular dysfunction in cerebral amyloid angiopathy. *Journal of Cerebral Blood Flow and Metabolism*. 2022;42:1272-1281. doi: doi:<https://dx.doi.org/10.1177/0271678X221076571>

18. Linortner P, Fazekas F, Schmidt R, Ropele S, Pendl B, Petrovic K, Loitfelder M, Neuper C, Enzinger C. White matter hyperintensities alter functional organization of the motor system. *Neurobiol Aging*. 2012;33:197.e191-199. doi: doi:10.1016/j.neurobiolaging.2010.06.005

19. Mascalchi M, Ginestroni A, Toschi N, Poggesi A, Cecchi P, Salvadori E, Tessa C, Cosottini M, De Stefano N, Pracucci G, et al. The burden of microstructural damage modulates cortical activation in elderly subjects with MCI and leuko-araiosis. A DTI and fMRI study. *Human Brain Mapping*. 2014;35:819-830. doi: doi:<https://dx.doi.org/10.1002/hbm.22216>

20. Nordahl CW, Ranganath C, Yonelinas AP, DeCarli C, Fletcher E, Jagust WJ. White matter changes compromise prefrontal cortex function in healthy elderly individuals. *Journal of Cognitive Neuroscience*. 2006;18:418-429. doi: doi:<http://dx.doi.org/10.1162/jocn.2006.18.3.418>

21. Papma JM, Den Heijer T, De Koning I, Mattace-Raso FU, Van Der Lugt A, Van Der Lijn F, Van Swieten JC, Koudstaal PJ, Smits M, Prins ND. The influence of cerebral small vessel disease on default mode network deactivation in mild cognitive impairment. *NeuroImage: Clinical*. 2013;2:33-42. doi: doi:<https://dx.doi.org/10.1016/j.nicl.2012.11.005>

22. Patel MJ, Boada FE, Price JC, Sheu LK, Tudorascu DL, Reynolds ICF, Aizenstein HJ. Association of small vessel ischemic white matter changes with BOLD fMRI imaging in the elderly. *Psychiatry Research - Neuroimaging*. 2012;204:117-122. doi: doi:<https://dx.doi.org/10.1016/j.pscychresns.2012.09.006>

23. Peca S, McCreary CR, Donaldson E, Kumarpillai G, Shobha N, Sanchez K, Charlton A, Steinback CD, Beaudin AE, Fluck D, et al. Neurovascular decoupling is associated with severity of cerebral amyloid angiopathy. *Neurology*. 2013;81:1659-1665. doi: 10.1212/01.wnl.0000435291.49598.54

24. Williams RJ, Goodyear BG, Peca S, McCreary CR, Frayne R, Smith EE, Pike GB. Identification of neurovascular changes associated with cerebral amyloid angiopathy from subject-specific hemodynamic response functions. *J Cereb Blood Flow Metab*. 2017;37:3433-3445. doi: 10.1177/0271678X17691056

25. Switzer AR, McCreary C, Batool S, Stafford RB, Frayne R, Goodyear BG, Smith EE. Longitudinal decrease in blood oxygenation level dependent response in cerebral amyloid angiopathy. *Neuroimage Clin*. 2016;11:461-467. doi: 10.1016/j.nicl.2016.02.020

26. Switzer AR, Cheema I, McCreary CR, Zwiers A, Charlton A, Alvarez-Veronesi A, Sekhon R, Zerna C, Stafford RB, Frayne R, et al. Cerebrovascular reactivity in cerebral amyloid angiopathy, Alzheimer disease, and mild cognitive impairment. *Neurology*. 2020;95:E1333-E1340. doi: doi:<https://dx.doi.org/10.1212/WNL.0000000000010201>

27. van den Brink H, Kopczak A, Arts T, Onkenhout L, Siero JCW, Zwanenburg JJM, Hein S, Hubner M, Gesierich B, Duering M, et al. CADASIL affects multiple aspects of cerebral small vessel function on 7T-MRI. *Annals of neurology*. 2022. doi: doi:<https://dx.doi.org/10.1002/ana.26527>

28. Vasudev A, Firbank MJ, Gati JS, Ionson E, Thomas AJ. BOLD activation of the ventromedial prefrontal cortex in patients with late life depression and comparison participants. *International Psychogeriatrics*. 2018;30:629-634. doi: doi:<https://dx.doi.org/10.1017/S1041610217000461>

29. Venkatraman VK, Aizenstein H, Guralnik J, Newman AB, Glynn NW, Taylor C, Studenski S, Launer L, Pahor M, Williamson J, et al. Executive control function, brain activation and white matter hyperintensities in older adults. *NeuroImage*. 2010;49:3436-3442. doi: doi:<https://dx.doi.org/10.1016/j.neuroimage.2009.11.019>

30. Salthouse TA. The role of memory in the age decline in digit-symbol substitution performance. *J Gerontol*. 1978;33:232-238. doi: 10.1093/geronj/33.2.232

31. Schroeter ML, Cutini S, Wahl MM, Scheid R, Yves von Cramon D. Neurovascular coupling is impaired in cerebral microangiopathy--An event-related Stroop study. *Neuroimage*. 2007;34:26-34. doi: doi:10.1016/j.neuroimage.2006.09.001

32. Hund-Georgiadis M, Ballaschke O, Scheid R, Norris DG, von Cramon DY. Characterization of cerebral microangiopathy using 3 Tesla MRI: correlation with neurological impairment and vascular risk factors. *J Magn Reson Imaging*. 2002;15:1-7. doi: 10.1002/jmri.10039

33. MacLeod CM. Half a century of research on the Stroop effect: an integrative review. *Psychol Bull*. 1991;109:163-203. doi: 10.1037/0033-2909.109.2.163

34. Tak S, Yoon SJ, Jang J, Yoo K, Jeong Y, Ye JC. Quantitative analysis of hemodynamic and metabolic changes in subcortical vascular dementia using simultaneous near-infrared spectroscopy and fMRI measurements. *NeuroImage*. 2011;55:176-184. doi: doi:<https://dx.doi.org/10.1016/j.neuroimage.2010.11.046>

35. Videbech P, Ravnkilde B, Gammelgaard L, Egander A, Clemmensen K, Rasmussen NA, Gjedde A, Rosenberg R. The Danish PET/depression project: performance on Stroop's test linked to white matter lesions in the brain. *Psychiatry Res*. 2004;130:117-130. doi: doi:10.1016/j.pscychresns.2003.10.002

36. Jokumsen-Cabral A, Aires A, Ferreira S, Azevedo E, Castro P. Primary involvement of neurovascular coupling in cerebral autosomal-dominant arteriopathy with subcortical infarcts and leukoencephalopathy. *J Neurol*. 2019;266:1782-1788. doi: doi:10.1007/s00415-019-09331-y

37. Wahlund LO, Barkhof F, Fazekas F, Bronge L, Augustin M, Sjogren M, Wallin A, Ader H, Leys D, Pantoni L, et al. A new rating scale for age-related white matter changes applicable to MRI and CT. *Stroke*. 2001;32:1318-1322. doi: 10.1161/01.str.32.6.1318

38. Lin WH, Hao Q, Rosengarten B, Leung WH, Wong KS. Impaired neurovascular coupling in ischaemic stroke patients with large or small vessel disease. *European Journal of Neurology*. 2011;18:731-736. doi: doi:<https://dx.doi.org/10.1111/j.1468-1331.2010.03262.x>

39. Monteiro A, Castro P, Pereira G, Ferreira C, Sorond F, Milstead A, Higgins JP, Polonia J, Azevedo E. Neurovascular Coupling Is Impaired in Hypertensive and Diabetic Subjects Without Symptomatic Cerebrovascular Disease. *Frontiers in Aging Neuroscience*. 2021;13:728007. doi: doi:<https://dx.doi.org/10.3389/fnagi.2021.728007>

40. Smith EE, Vijayappa M, Lima F, Delgado P, Wendell L, Ros, J, Greenberg SM. Impaired visual evoked flow velocity response in cerebral amyloid angiopathy. *Neurology*. 2008;71:1424-1430. doi: doi:<https://dx.doi.org/10.1212/01.wnl.0000327887.64299.a4>

41. Sorond FA, Hurwitz S, Salat DH, Greve DN, Fisher ND. Neurovascular coupling, cerebral white matter integrity, and response to cocoa in older people. *Neurology*. 2013;81:904-909. doi: doi:10.1212/WNL.0b013e3182a351aa

42. Sorond FA, Kiely DK, Galica A, Moscufo N, Serrador JM, Iloputaife I, Egorova S, Dell'Oglio E, Meier DS, Newton E, et al. Neurovascular coupling is impaired in slow walkers: the MOBILIZE Boston Study. *Ann Neurol*. 2011;70:213-220. doi: 10.1002/ana.22433

43. Daselaar SM, Veltman DJ, Rombouts SA, Raaijmakers JG, Jonker C. Neuroanatomical correlates of episodic encoding and retrieval in young and elderly subjects. *Brain*. 2003;126:43-56. doi: 10.1093/brain/awg005
